# When Matter Builds Itself: Blueprints from Life to Materials

**DOI:** 10.1021/acs.nanolett.6c00057

**Published:** 2026-04-02

**Authors:** L. Aloisio, F. Marangi, G. Lanzani

**Affiliations:** † Laboratory of Organic Electronics, Department of Science and Technology, Linköping University, SE-60174 Norrköping, Sweden; ‡ Center for Nanoscience and Technology, Istituto Italiano di Tecnologia, Via Rubattino 81, 20134 Milan, Italy; § Department of Physics, Politecnico di Milano, Piazza Leonardo da Vinci 32, 20133 Milan, Italy

**Keywords:** supramolecular materials, intracellular nanostructures, self-assembly in living systems, organic semiconductors, bioelectronic interfaces

## Abstract

Self-assembly is pervasive in living systems but remains an unconventional paradigm for constructing functional materials in biological environments. Rather than relying on ex situ fabrication and subsequent integration, in situ self-assembly enables the autonomous formation of supramolecular architectures under physiological conditions, allowing synthetic materials to couple directly with biological functions. This Mini-Review surveys recent advances in functional self-assembly across intracellular, extracellular, and microbial realms, focusing on materials-driven strategies that introduce new electrical, optical, and mechanical capabilities into living systems. Examples range from intracellular semiconducting nanostructures and optically active supramolecular assemblies to extracellular conductive interfaces and engineered microbial materials. Together, these studies establish self-assembly as an active mechanism for augmenting biological function and a scalable route toward biointegrated, adaptive materials.

## Graphical abstract

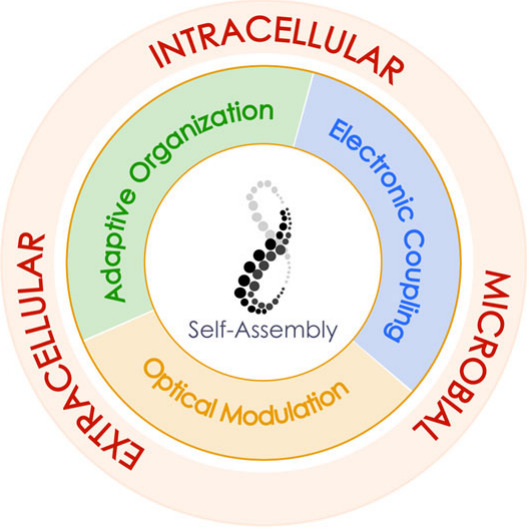

Although self-assembly is fundamental and pervasive in nature, it is not the standard paradigm in materials science and technology. Devices are typically fabricated via multistep, top-down process flows under tight external control.^1^ Consequently, achieving functionality through self-assembly remains uncommon and distinctive, despite providing a powerful strategy for constructing materials with molecular precision.^2,3^ The concept of self-assembly can be extended from controlled laboratory experiments into living systems, where assembly occurs directly within the complex and dynamic cellular environment.^4^ Indeed, countless cellular, tissue, and microbial structures demonstrate that self-assembly is an intrinsic construction route for functional living systems, for example phospholipids spontaneously organize into bilayers, endothelial cells form capillary-like networks, and *Bacillus subtilis* develops self-organized fractal colonies under nutrient stress.^5–7^

From a materials-science perspective, living environments impose a unique set of design constraints, requiring assemblies to form and persist under physiological conditions.^4^ Yet these same constraints create opportunities by providing endogenous triggers and localized cues which can be exploited to drive the autonomous formation of nanofibers, condensates, hydrogels and other supramolecular architectures.^8^ Possible mechanistic pathways include noncovalent supramolecular interactions, enzymatically triggered assembly or covalent network formation, redox driven processes including oxidative polymerization, and stimulus mediated assembly driven by physical inputs such as light, electric fields, or mechanical forces. In this setting, *in situ* self-assembly enables structures that do not merely coexist with biological matter but actively restore impaired functions or introduce entirely new capabilities, leveraging emergent properties transcending those of their individual isolated components and allowing functions to arise from supramolecular architecture rather than chemical composition.^9^

In this mini-review, we highlight recent advances in functional self-assembly within living environments, emphasizing materials-driven strategies that achieve added or restored biological functionality. By focusing on intracellular, extracellular and microbial contexts, we outline how self-assembly can be used as a mode of augmentation, transforming biological systems through the programmed construction of exogenous functional supramolecular structures (Figure 1). This approach could enable minimally invasive, adaptive, and electronically active biointegrated materials and devices.

**Figure 1:**
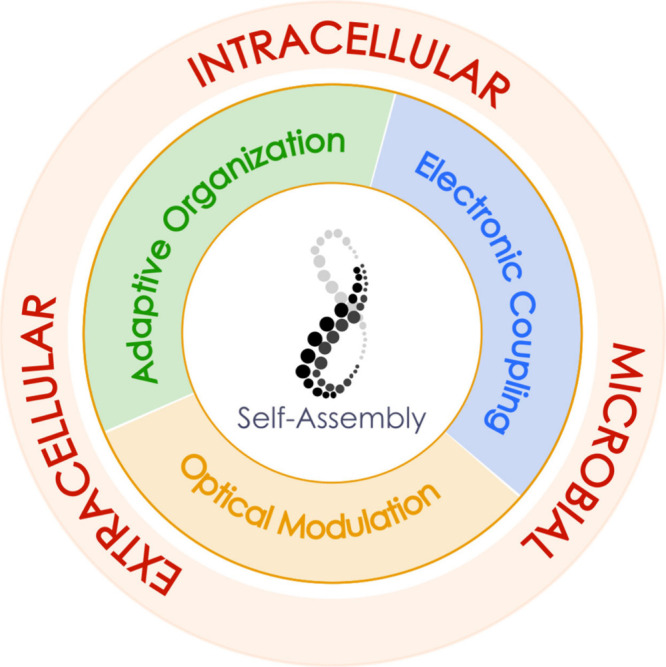
Conceptual framework of functional self-assembly in living systems. Self-assembly is depicted as a central mechanism enabling the formation of supramolecular architectures with emergent physical functions. The middle ring highlights the principal functional outcomes arising from materials-driven self-assembly processes discussed in this Review: electronic coupling, optical modulation, and adaptive organization. The outer ring indicates the biological context in which these strategies operate, spanning intracellular, extracellular, and microbial systems. The diagram emphasizes how self-assembly provides a unifying route to introduce new physical functionalities across biological scales.

## Intracellular Self-Assembly

Integrating functional materials and devices into living systems enables novel and efficient approaches to interrogation and stimulation. However, many materials are chemically synthesized or assembled *ex vivo* prior to integration, which creates substantial challenges for their subsequent incorporation into the biological environment. Intracellular self-assembly at the micro- and nanoscale bypasses this limitation.^10^ Many functional assemblies derive from peptides and enzymatic activity, which can promote both noncovalent self-assembly and covalent network formation. This topic is well represented in literature and has been thoroughly reviewed.^11,12^ Additionally, extensive work has explored the use of self-assembled structures to manipulate cell fate, with particular emphasis on inducing apoptosis or inhibiting proliferation in cancer cells.^13^ While many studies have focused on biochemical outcomes, emerging work shows that intracellular assemblies can also impart new physical functions.

### Electronic Enhancements

Electrical phenomena are central to cellular functions, governing processes such as membrane excitability, ion transport, and signal propagation. In living systems, electrical activity is almost exclusively mediated by ions, and it is tightly confined by membranes and subcellular compartmentalization. Therefore, electrical interactions are highly localized and rapidly dissipated, limiting direct coupling between distinct intracellular regions or across neighboring cells.^14^ While this mode of operation is essential for physiology, it also limits the ways in which electrical interactions can be accessed, recorded, or redistributed within and among cells or tissues. The introduction of electronic charge transport into this landscape would enable a fundamentally different mode of interaction, based on mixed electronic-ionic transport, allowing electrical activity to extend beyond native biological constraints.

A clear example of intracellular electronic augmentation is provided by lycobetaine nanofibers, which spontaneously form inside living cells through supramolecular self-assembly initiated by a transient temperature drop that shifts solubility and local supersaturation, enabling rapid nucleation of nanofibers (Figure 2A,B).^15^ Upon internalization, lycobetaine (LBT) organizes into elongated, wirelike structures that align along the mitochondrial network (Figure 2C) without compromising cell viability. These intracellular assemblies display semiconducting behavior, characterized by nonlinear current–voltage responses, indicating the presence of electronic charge transport within the fibers. The formation of these nanocables establishes physical coupling between mitochondria, connecting spatially separated organelles and synchronizing their membrane potential dynamics across the cell (Figure 2D), which cannot be achieved through native ionic signaling alone.^15^

**Figure 2:**
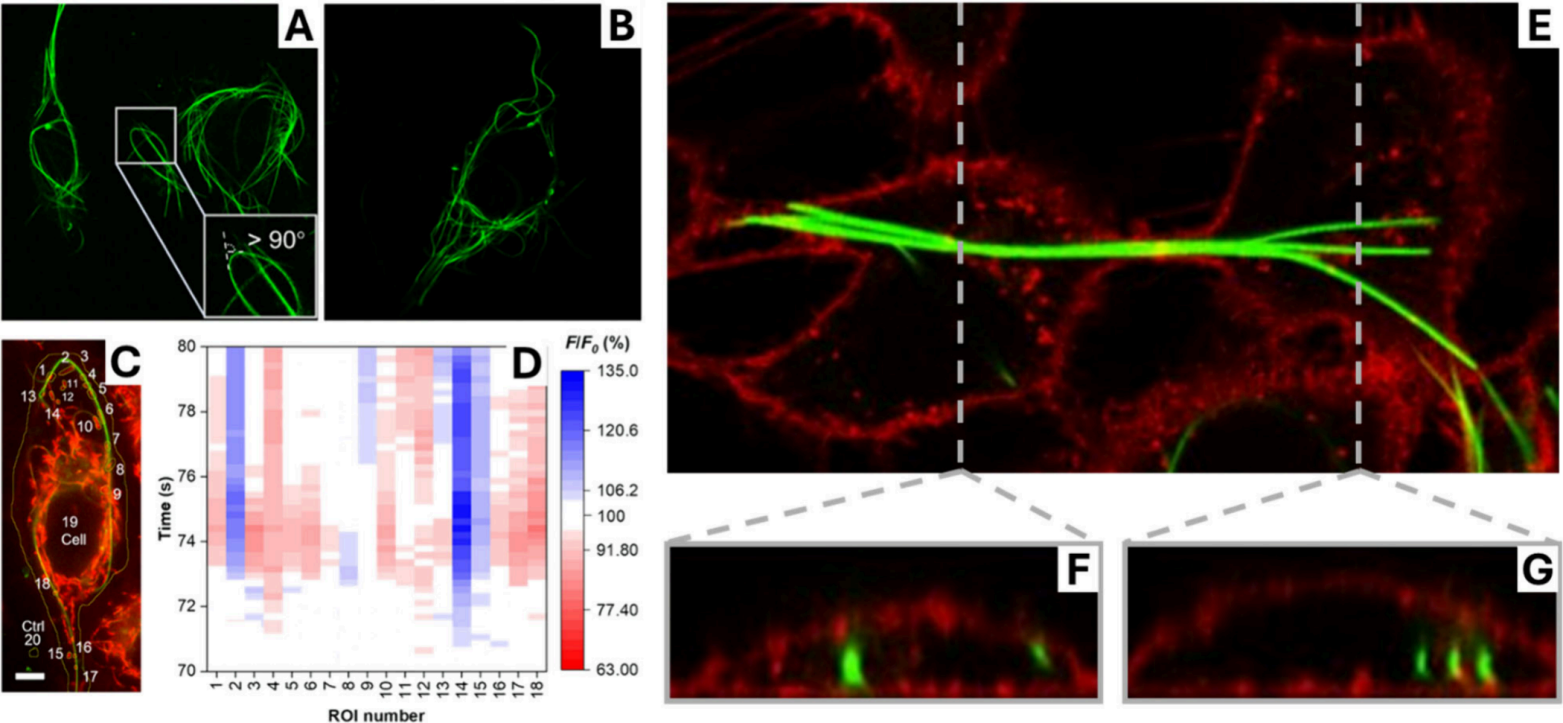
Semiconducting Nanofibers enable intra- and intercellular connectivity (A, B) Confocal fluorescence images showing intracellular LBT nanocables formed in living cells following a transient temperature drop. Adapted from ref 15. (C) Mitochondrial network labeled in red, with numbered regions of interest (ROIs) used for quantitative analysis of membrane potential dynamics. Adapted from ref 15. (D) Heat map of mitochondrial membrane potential fluctuations across ROIs, revealing synchronized dynamics between spatially separated mitochondria connected by LBT fibers. Adapted from ref 15. (E) Confocal image showing supramolecular fibers formed by the conjugated thiophene derivative DTTO (green) within and across adjacent cells (red, membrane stain). DTTO fibers remain mechanically compliant and integrate with dynamic cellular processes. Adapted from ref 18. (F, G) Section of Z-stack cut along the corresponding vertical directions, orthogonal to the fibers. Adapted from ref 18.

While intracellular electronic augmentation already represents a departure from native biological signaling, an even more significant step could be achieved by self-assembled electronic structures that extend beyond the boundaries of a single cell. This behavior has been observed for the conjugated thiophene derivative DTTO, which spontaneously forms supramolecular fibers inside living cells under physiological conditions (Figure 2E).^16^ This behavior is consistent with a nonclassical crystallization route, where DTTO first organizes into disordered clusters or liquid like precursors promoted by differences in solubility across intracellular compartments, and fibers nucleate from these intermediates.^17^ Importantly, these elongated assemblies are neither rigid nor disruptive: fibers remain mechanically compliant and integrate with dynamic cellular processes, for instance consistently deforming within contracting C2C12 myotubes.^18^ Furthermore, observations of DTTO fiber formation in vivo indicate that the same self-assembly process can occur in more complex biological environments, supporting its scalability beyond isolated cell systems.^19^

Similarly to LBT fibers, electrical and spectroscopic measurements indicate that these intracellular fibers exhibit semiconducting behavior, supporting electronic charge transport. Rather than remaining confined within individual cells, DTTO fibers can pierce and extend across cell membranes, spanning adjacent cells, and forming continuous structures that physically connect intracellular environments of distinct cells (Figure 2E-G).^18^ In this way, DTTO self-assembly introduces electronic continuity across multiple cells, a situation that has no direct analogue in native biological systems, where electrical communication is mediated indirectly through ionic signaling and membrane bound proteins.

### Optical Enhancements

Light interacts with living systems in different ways, depending on the properties of cellular absorbers, providing functional advantages in some organisms, e.g. orientation in space, energy fueling or vision, while acting as a source of stress in others, e.g. reactive oxygen species (ROS) generation. In many cellular systems, however, light does not play a direct biological role, and its interaction with matter remains largely uncontrolled.^20^ Introducing optically driven functionalities via self-assembled materials offers a route to control cells behavior with light. To this end, supramolecular structures enable the tailoring of optical responses by leveraging intermolecular coupling and excitonic (wave function) delocalization. This can give rise to collective states with engineered properties, such as enhanced radiative decay, increased absorption cross-section, tunable optical gaps, and polarized emission. Functional outcomes may include photoprotection, optical interrogation via intracellular light-emitting or even lasing components used as cellular tags, and light-driven modulation of cellular activity.

A representative example is provided by guanine crystals in the eyes of zebrafish, where intracellular reflective structures contribute to directional control of light (Figure 3A–D).^21^ These photonic elements form through the intracellular self-assembly of guanine molecules, which nucleate and crystallize within membrane bound vesicles whose pH varies during maturation, enabling guanine accumulation at early stages and promoting nucleation and growth as the pH shifts.^22^ Recent work has shown that this process is regulated by the local intracellular environment, leading to the growth of plate-like crystalline assemblies with defined orientation and optical function, without the involvement of preformed templates or scaffolds. By exploiting these compartments, similar architectures could be recreated in systems that are highly sensitive to solar irradiation, such as corals, where excessive light contributes to photodamage and bleaching.^23^

**Figure 3:**
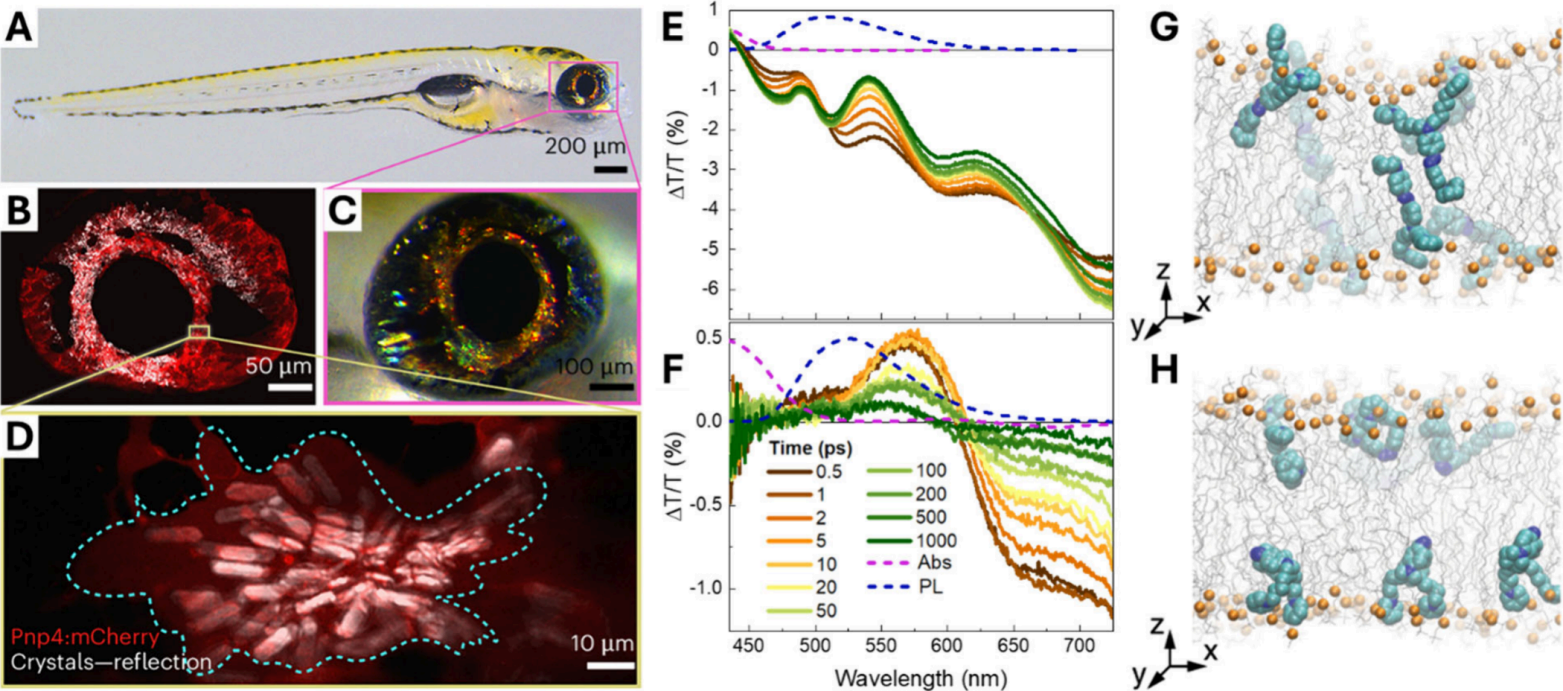
Natural and engineered routes to intracellular optical function (A–D) Guanine molecules nucleate and crystallize within membrane-bound intracellular compartments, forming plate-like crystalline assemblies with defined orientation. Panels show representative whole-organ, cellular, and subcellular views highlighting the hierarchical organization and optical response of the guanine-based structures. Adapted from ref 22. (E–F) Transient absorption measurements of (E) molecular DTTO and (F) DTTO supramolecular fibers revealing excited-state dynamics and signatures of optical gain arising from collective photophysical behavior within the supramolecular assemblies. Adapted from ref 29. (G–H) Molecular dynamics simulations of the amphiphilic azobenzene derivative Ziapin2 embedded in a lipid bilayer illustrating molecular partitioning and dimer formation across membrane leaflets, which underlies light-induced modulation of membrane properties. Adapted from ref 30.

While naturally occurring crystalline assemblies, such as guanine platelets, primarily serve passive optical components, the same principles of intracellular organization can be extended toward active optical functionalities. This conceptual transition from endogenous photonic structures toward engineered supramolecular assemblies capable of generating or amplifying optical signals sets the stage for the use of self-assembled materials as intracellular optical probes and light-based interrogation strategies.

Optical interrogation of living cells has been demonstrated using intracellular light emitting and light confining structures that act as optical tags.^24^ Micro or nanoscale resonators introduced into cells enable narrowband emission or lasing under optical excitation, providing multiplexing optical signatures for cell tracking and identification (up to thousand different channels).^25,26^ In addition, intracellular light sources can enable local imaging of organelles alternative to traditional microscopy.^27^ Self-assembled organic materials offer an alternative route to achieve similar functionalities directly inside living cells: supramolecular structures formed in situ can display collective optical properties that do not arise at the molecular level.^28^ For example, DTTO fibers exhibit strong stimulated emission under optical excitation, indicating their ability to sustain optical gain (Figure 3E,F).^29^ When combined with geometric confinement of light along elongated supramolecular architectures, such properties create the conditions required for lasing within the intracellular environment.^28^ In this framework, these structures could act as intracellular optical tags, analogous to previously demonstrated cell based microlasers, while being formed autonomously inside cells from small molecular building blocks, avoiding rigid prefabricated resonators.

Light is also a powerful tool for stimulating biological systems, as it can be delivered with high spatial and temporal precision, yet most cells do not natively convert optical stimuli into functional responses, leaving a largely untapped opportunity for active modulation.^20^ A representative example is provided by the amphiphilic azobenzene derivative Ziapin2, which self-assembles into dimers within the plasma membrane, with the two molecules embedded in opposite leaflets (Figure 3G,H).^30^ In the dark, dimer formation locally reduces membrane thickness without inducing functional effects. Upon illumination, photoisomerization disrupts the dimeric assembly, allowing the membrane to relax back to its original thickness, thus inducing a transient change in membrane capacitance that can trigger action potentials in excitable cells.^31^ In addition, light-induced disruption of the supramolecular assembly can generate a strain wave in the membrane that gates mechanosensitive ion channels – an effect that is particularly relevant in contracting cells such as skeletal muscle cells and cardiomyocytes.^32^

## Extracellular Self-Assembly

While intracellular self-assembly relies on mild, noncovalent interactions, the extracellular environment allows access to a broader range of chemical processes. Reactions such as oxidative polymerization, which would be difficult to control or potentially disruptive inside the cell, can instead be exploited outside the plasma membrane to generate functional electronic materials directly at the cell interface.^33^

In situ formation of conductive organic materials within biological environments has been achieved through biologically driven oxidative polymerization of thiophene-based monomers, where enzymatic or metabolically generated oxidants catalyze material growth under physiological conditions. Polymerization of ETE-S on native lipid membranes (Figure 4A), which represent a model of the extracellular interface, proceeds via horseradish peroxidase (HRP) in the presence of hydrogen peroxide (H_2_O_2_), yielding thin conductive coatings that adhere to mammalian-derived bilayers without disrupting their integrity.^34^ Building on this concept, thiophene derivatives bearing membrane-anchoring moieties were designed to insert into lipid bilayers and support polymer growth extracellularly, directly at the cell surface, while preserving viability and calcium signaling (Figure 4B). The resulting cell-bound domains exhibited conductivities consistent with mixed ionic/electronic transport characteristics of organic bioelectronic materials (Figure 4C–D).^35^ These results show that oxidative polymerization can be spatially confined to the extracellular space and proceed at the single-cell level in a biocompatible manner, producing stable interfaces capable of direct electrical interaction with living cells.

**Figure 4:**
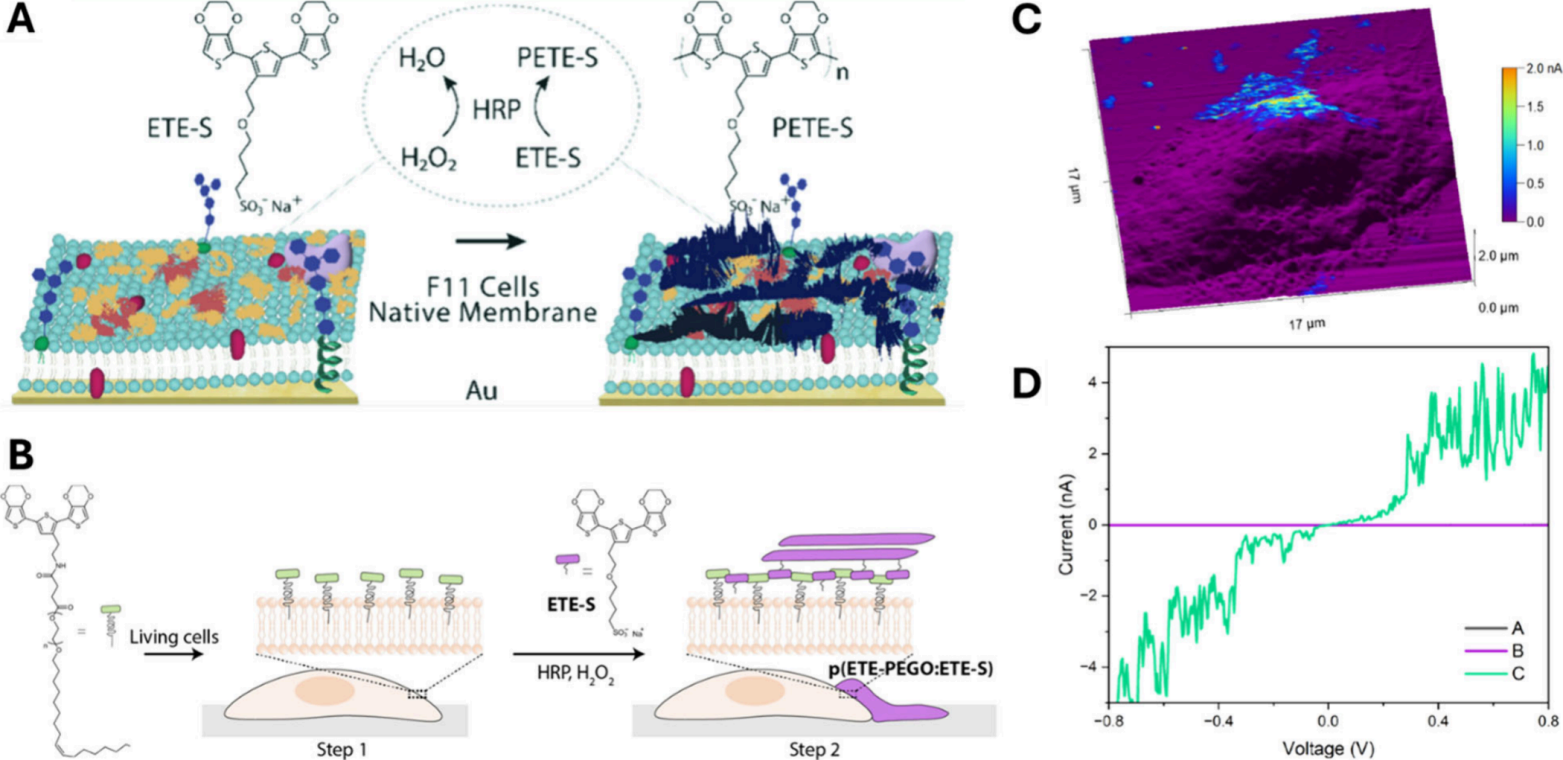
In situ polymerization generates conductive interfaces at living cell membranes. (A) Schematic native lipid membrane, showing the ETE-S monomer (yellow) polymerized to form PETE-S (black) catalyzed by the enzyme HRP (orange) in the presence of H_2_O_2_ as oxidant; the chemical structures of the monomer and polymer are shown above. Adapted from ref 34. (B) Schematic overview of polymer anchoring on living cells. First, the anchor molecule (ETE-PEGO) is introduced in the membranes, after insertion of the anchor molecule, the enzyme (HRP), catalyst (H_2_O_2_), and monomer (ETE-S) are added to start polymerization. Adapted from ref 35. (C) Topography image of cells coated by PETE-S obtained by conductive AFM, showcasing the presence of a conductive region on top of the cell. Adapted from ref 35. (D) I–V curves measured on different points by conductive AFM: on substrate (black line), on coating polymerized directly on the cell (green line), and on uncoated regions of the cell (purple line). Adapted from ref 35.

The same chemistry was subsequently extended in vivo, where endogenous metabolites and peroxidases provide the redox environment required for polymerization. In the zebrafish brain and along the leech nerve cord, metabolic activity drives the emergence of soft, tissue-conformal conductive networks that follow local tissue morphology while sustaining neural signaling. Under these conditions, the bioformed conductors were sufficiently functional to support active neuromodulation, as demonstrated by reversible muscle contractions in leech upon electrical stimulation through the in situ generated material.^36^

## Harnessing Microbial Communities for Material Functionality

The examples above illustrate how intracellular and extracellular assemblies can endow eukaryotic cells and tissues with new electrical and optical functionalities. Microbial systems, however, offer a complementary landscape in which both intrinsic cellular organization and engineered interfaces can yield functional material behaviors. Bacteria can be regarded as micromachines capable of performing device-relevant functions, such as electron transport or charge injection at electrodes. Moreover, their motility enables mass transport and exploration of the surrounding environment. Engineering bacteria with self-assembled nanoarchitectures that can harness and direct these functions may have important implications for biohybrid technologies. DNA-based architectures provide fully abiotic and programmable paths to intracellular structure formation, through sequence encoded molecular recognition, enabling the regulation of organelles, sensing of intracellular targets, and modulation of cellular behavior in mammalian cells.^37^ In situ polymerization of synthetic monomers likewise produces functional polymer networks directly inside living cells, via covalent network formation that can be supported by cellular redox chemistry or enzymes, demonstrating that abiotic chemistries can also operate in the intracellular environment to reconfigure cellular behavior.^38^ In *Escherichia coli*, genetically encoded single-stranded DNA motifs can autonomously assemble into nanostructures in vivo, showing that bacterial cells can serve as chassis for programmable supramolecular architectures.^39^ These examples collectively highlight the breadth of intracellular assembly mechanisms and underscore their potential to operate across biological kingdoms, forming a natural transition toward larger-scale constructs found in the extracellular environment. The integration of exogenous materials with microbial cells extends these concepts into engineered contexts where abiotic structures directly augment biological functions. Several strategies rely on the functionalization of microbial surfaces through interface engineering. As an example, materials-mediated functions including enhanced stability, targeted delivery, and therapeutic activity can be achieved through surface nanocoatings.^40^ Lipid or polymeric coatings directly assembled on bacterial cells, largely driven by interfacial noncovalent interactions and confinement at the surface, act as protective and functional layers enabling prolonged survival and efficient delivery in physiological environments (Figure 5A, B).^41^

**Figure 5:**
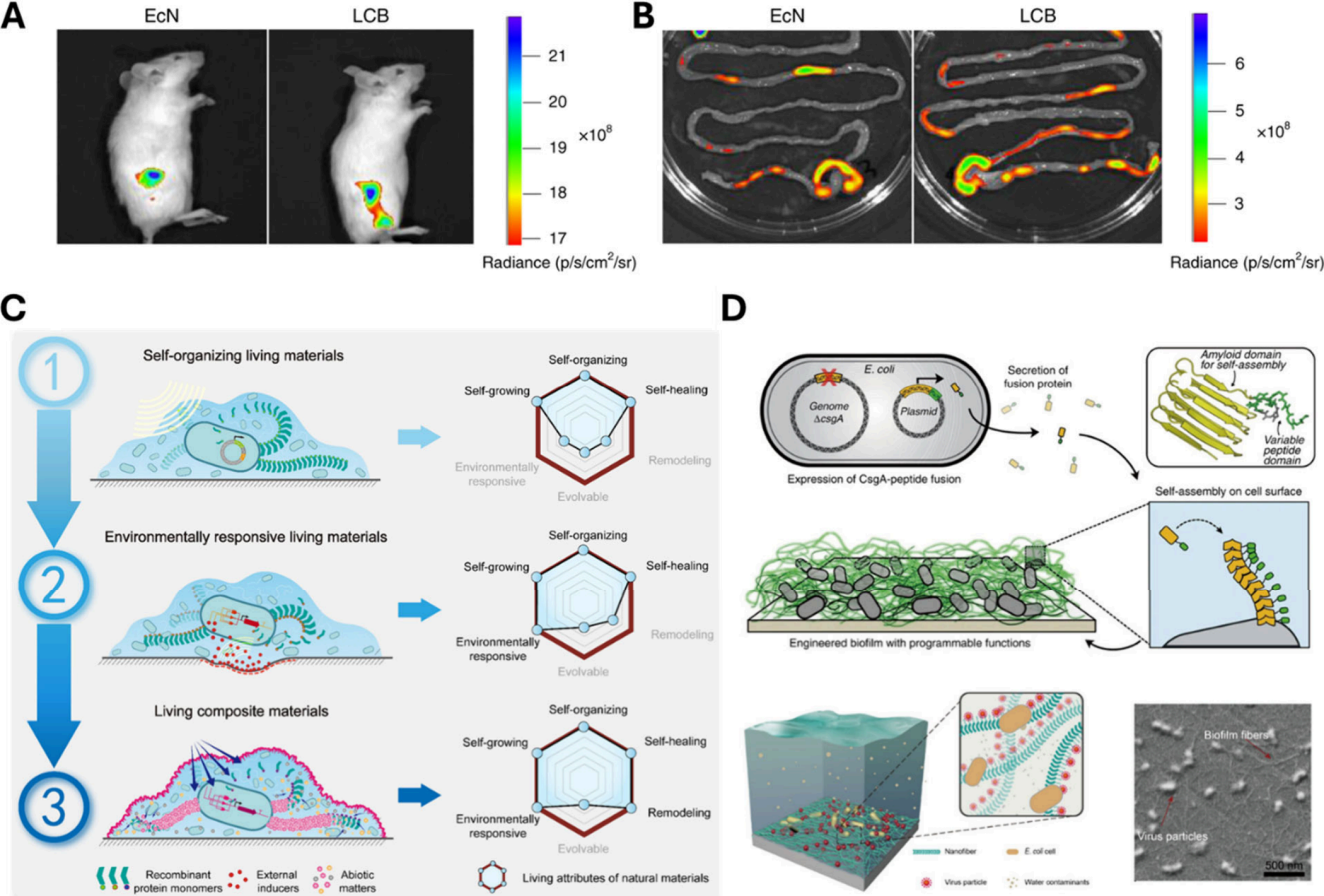
Materials-enabled functional microbial systems from interfaces to living materials. Protective self-assembled lipid layers allow increased survival of bacteria in the gastrointestinal tract. Representative IVIS image of mice 4 h (A) and their intestinal tracts (B) after oral gavage of eGFP-expressing uncoated EcN and LCB. Adapted from ref 41. (C) Three types of engineered living materials (ELM) based on bacterial biofilms. The progression from natural living materials to ELM incorporating both biological and synthetic exogenous elements enables functional enhancements for improved performance. Reprinted from ref 42. (D) Representative living materials based on *Escherichia coli* curli biofilms able to secrete amyloid CsgA fused with versatile, functional protein domains (top). Engineered *E. coli* biofilms-enabled disinfection of the influenza virus from river water (bottom). Reprinted from ref 42

Yet, augmenting microbial function does not rely solely on exogenous materials: the innate organizational behaviors of bacteria themselves can be leveraged to create engineered living materials, in which collective cellular organization becomes a design element (Figure 5C,D).^42^ Those naturally self-organized bacterial communities in an extracellular matrix can be rewired with synthetic or hybrid components, still preserving inherent self-assembly while developing new capabilities such as antibiotic resistance, catalytic activity or responsiveness to environmental cues.^43^ Together, these approaches demonstrate that bacteria can act as both building blocks and active components of materials with functional outputs not accessible to native cells alone.

Beyond bacterial systems, unicellular microorganisms such as diatoms can autonomously generate hierarchically structured inorganic architectures with optical and mechanical functionality, highlighting biological fabrication routes in which structure emerges in vivo even when functional deployment often occurs ex situ.^44^

## Functional Implications and Outlook

From intracellular assemblies to extracellular interfaces, self-organization provides a continuous spectrum of integration between synthetic materials and living systems. Across these scales, assembly is no longer merely a means of constructing structure but becomes a mechanism through which molecular information is translated into biological function. By exploiting endogenous biochemical activity, confinement, and dynamic cellular environments, functional materials can emerge in situ in forms that are adaptive, localized, and inherently compatible with living matter. At cellular interfaces, strategies such as oxidative polymerization of thiophene-based monomers enable the formation of conductive coatings that conform to native membranes while preserving cellular viability. More broadly, supramolecular systems based on noncovalent interactions establish that dynamic and bioactive architectures can form without rigid scaffolds or invasive processing, offering flexible routes to functional biointerfaces. Within cells, ordered assemblies further extend this paradigm by unlocking physical properties that are inaccessible to native biomolecules alone. Supramolecular fibers, crystalline semiconductors, and membrane-bound molecular systems allow electronic, optical, and optoelectronic functions to emerge from spatial organization rather than chemical composition.

At larger biological scales, microbial systems extend these concepts by coupling intrinsic cellular self-organization with engineered material interfaces. Bacteria can act both as active building blocks and as organizational scaffolds, giving rise to living or hybrid materials in which collective behavior, metabolic activity, and material function become intertwined. Strategies based on surface functionalization, interfacial self-assembly, or the rewiring of naturally self-organized microbial communities demonstrate how adaptive, resilient, and multifunctional material behaviors can emerge without imposing rigid external architectures.

Taken together, these advances position self-organization as a functional transduction principle, converting molecular design into emergent optical, electrical or adaptive behaviors within living systems. The observation of both polymerization- and supramolecular assembly-driven material formation in vivo further indicates that these processes are not restricted to simplified model systems, but can operate across cells, tissues and microbial communities. In this context, assembly also becomes a strategy for localization: rather than delivering preformed materials, simple molecular precursors can be guided to specific intracellular or interfacial targets, where functionality emerges only upon organization.

Looking ahead, a central challenge lies in developing predictive frameworks that link molecular structure to assembly pathways, spatial localization, stability, and functional output within complex biological environments. Achieving this will require deeper insights into how local biological cues (redox gradients, enzymatic activity, mechanical forces, and confinement) shape material organization in living systems. Progress in this direction could enable a new generation of efficient self-assembled functional hybrids with unprecedented capabilities, able to coexist and cofunction with biological components. Such systems may ultimately underpin theranostic and biointerfacing platforms in which material formation, targeting and function are intrinsically coupled, paving the way toward living materials with capabilities extending beyond those achievable by either synthetic or biological components alone.
